# The Global Economic and Health Burden of Human Hookworm Infection

**DOI:** 10.1371/journal.pntd.0004922

**Published:** 2016-09-08

**Authors:** Sarah M. Bartsch, Peter J. Hotez, Lindsey Asti, Kristina M. Zapf, Maria Elena Bottazzi, David J. Diemert, Bruce Y. Lee

**Affiliations:** 1 Public Health Computational and Operational Research (PHICOR), Johns Hopkins Bloomberg School of Public Health, Baltimore, Maryland, United States of America; 2 Global Obesity Prevention Center, Johns Hopkins Bloomberg School of Public Health, Baltimore, Maryland, United States of America; 3 National School of Tropical Medicine, and Departments of Pediatrics and Molecular Virology & Microbiology, Baylor College of Medicine, Houston, Texas, United States of America; 4 Sabin Vaccine Institute, Washington, D.C., United States of America; 5 Department of Microbiology, Immunology and Tropical Medicine, The George Washington University Medical Center, Washington, D.C., United States of America; University of Kelaniya, SRI LANKA

## Abstract

**Background:**

Even though human hookworm infection is highly endemic in many countries throughout the world, its global economic and health impact is not well known. Without a better understanding of hookworm’s economic burden worldwide, it is difficult for decision makers such as funders, policy makers, disease control officials, and intervention manufacturers to determine how much time, energy, and resources to invest in hookworm control.

**Methodology/Principle Findings:**

We developed a computational simulation model to estimate the economic and health burden of hookworm infection in every country, WHO region, and globally, in 2016 from the societal perspective. Globally, hookworm infection resulted in a total 2,126,280 DALYs using 2004 disability weight estimates and 4,087,803 DALYs using 2010 disability weight estimates (excluding cognitive impairment outcomes). Including cognitive impairment did not significantly increase DALYs worldwide. Total productivity losses varied with the probability of anemia and calculation method used, ranging from $7.5 billion to $138.9 billion annually using gross national income per capita as a proxy for annual wages and ranging from $2.5 billion to $43.9 billion using minimum wage as a proxy for annual wages.

**Conclusion:**

Even though hookworm is classified as a neglected tropical disease, its economic and health burden exceeded published estimates for a number of diseases that have received comparatively more attention than hookworm such as rotavirus. Additionally, certain large countries that are transitioning to higher income countries such as Brazil and China, still face considerable hookworm burden.

## Author Summary

Our work quantifies the economic and health burden associated with hookworm infection by country, WHO region, and worldwide. We also evaluated the burden by age, various costing methodologies, and ran an extensive sensitivity analysis on key and debated parameters to determine the robustness of our model’s resulting estimates. We report the DALYs associated with hookworm infections with consequent health outcomes in 2016 and show the impact that varying disability weights and likelihoods of anemia have on the estimates. Additionally, our study identified potential targets for future studies and data collection. Our results provide important information for decision makers. Without a better understanding of the economic and health burden of hookworm worldwide and in different countries, it is difficult for decision makers such as funders, policy makers, disease control officials, and intervention manufacturers to determine how much time, energy, and resources to invest in hookworm control. Our results can also help decision makers know where hookworm should fall on their priority lists and allocate limited resources.

## Introduction

Even though human hookworm infection (hookworm) is highly endemic in many low- and middle-income countries throughout the world, its global economic and health impact is not well known. Unlike other soil-transmitted helminth (STH) infections (e.g., ascariasis and trichuriasis), high intensity hookworm infection commonly affects both children and adults.[1, 2] Hookworm is typically controlled through mass drug administration (MDA) programs. While these programs have successfully reduced morbidity due to STHs among children, it is not having a similar effect on hookworm. In fact, MDA with mebendazole is not reducing the prevalence of hookworm-related anemia[3], while the impact of MDA with albendazole in children is inconsistent[4, 5]. Moreover, the Global Burden of Disease (GBD) Study 2013 estimates that MDA is not having a significant effect in reducing in the prevalence of hookworm infection.[6] Even when MDA programs are in place they primarily target pre-school and school-aged children[7, 8], leaving high-burden groups untreated.

Despite these control programs, hookworm’s disease burden remains high, currently, hookworm affects approximately 500 million people[6], with 5.1 billion at risk for acquiring infection worldwide.[9] However, hookworm infection rarely results in death, but instead leads to iron-deficiency anemia and malnutrition[10], which cause more subtle chronic health problems such as lethargy, impaired physical and cognitive development, and adverse pregnancy outcomes[1]. Therefore, measures that focus on severe disease outcomes such as mortality and hospitalization will severely underestimate the economic and health burden of hookworm. Moreover, those infected with hookworm may have concomitant health conditions, such as malaria infection, that can also cause anemia.[11] Without a better understanding of the economic and health burden of hookworm worldwide and in different countries, it is difficult for decision makers such as funders, policy makers, disease control officials, and intervention manufacturers to determine how much time, energy, and resources to invest in hookworm control. Therefore, we developed a computational simulation model to meet this need.

## Methods

### Model Structure

Using Microsoft Excel (Microsoft, Redmond, WA) along with a Crystal Ball (Oracle, Redwood City, CA) add-in, we developed a Monte Carlo simulation model to determine the economic and health burden of hookworm infection by country (N = 159, all countries where prevalence is reported and transmission is viable), WHO region, and worldwide from the societal perspective. The model converted age-specific hookworm prevalence and population estimates of different locations into hookworm cases by age, determined the intensity level of each hookworm infection, converted each case into health outcomes, and translated health outcomes into productivity losses and disability-adjusted life years (DALYs).

The model starts with age-stratified hookworm prevalence estimates by location, using the following age strata (*i*): children 0–4 years old, 5–9 years old, 10–14 years old, and adults 15 years and older. We determined the infection intensity level by calculating the mean worm burden (M, average number of worms per person) of the age-stratified population from the prevalence and applied a negative binomial distribution of worms in the community[12] resulting in the probability of an individual harboring a given number of worms. The negative binomial distribution allowed us to represent the over-dispersion of worms, such that a few individuals harbor most of the worms. The model estimated M based on the hookworm prevalence (P) for each age-specific population using the following equation:

Mi=-k1-Pi1k-11-Pi-1k

derived from Anderson and May[12], where k is the degree of aggregation of worms within the human population (where the proportion of the human population harboring the majority of hookworms becomes smaller as k approaches 0), and was set at 0.34.[13, 14] We calculated the probability of low (<28 worms), moderate (28–65 worms), and heavy intensity (>65 worms) infection[15] by fitting M to the negative binomial distribution:

Pix=1+Mik-k-x*Mikx*Гk+xx!Гx

where Г() is a gamma distribution and x is the number of worms. These probabilities multiplied by the total population determined the number of persons in each age group with each infection intensity level (i.e., number of persons with low, moderate, or heavy intensity infections in each age group).

Our model then converted each hookworm infection into health outcomes (i.e., hookworm-associated anemia and cognitive impairment) with accompanying health effects and costs. Each person with a hookworm infection had a probability of having hookworm-associated anemia. We derived the probability of hookworm-associated anemia using the age-stratified, infection intensity-specific probability of anemia and population attributable fraction (PAF). Age- and intensity-specific probabilities of anemia among those with hookworm infection came from the literature (Table 1). While there is a direct relationship between the intensity of hookworm infection (i.e., number of adult worms inside the host) and host blood loss[16], hookworm is not the only cause of anemia. We used the PAF, or the proportion of anemia in the population that is due to hookworm (Table 1), to attenuate the probabilities of anemia to determine the probability of hookworm-associated anemia. Each person with anemia then had a probability of having a specific hemoglobin (Hb) level. This probability was pulled from an age- and infection intensity-specific distribution. The distribution of Hb levels for each infection intensity level and age group was custom and created using data extracted from the literature on Hb levels for those with anemia and a hookworm infection.[16–21] As infants and children are particularly vulnerable to developmental and behavioral deficits, most likely due to iron-deficiency anemia[1], only children (0–14 years old) with anemia due to moderate and heavy intensity infections were assumed to have cognitive impairment.

**Table 1:** Model input parameter values and sources.

Parameter	All Infection Intensities	Low Intensity Infection	Moderate Intensity Infection	Heavy Intensity Infection	Source
Disability Weights					
Anemia (2004 Estimate)	0.024				[49]
Heavy Intensity Infection (2004 Estimate)				0.006	[49]
Cognitive Impairment (2004 Estimate)^ǂ^			0.024	0.024	[49]
Mild Anemia (2010 Estimate)†	0.005 (0.002–0.011)				[50]
Moderate Anemia (2010 Estimate)†	0.058 (0.0380–0.086)				[50]
Severe Anemia (2010 Estimate)†	0.164 (0.112–0.228)				[50]
Symptomatic Intestinal Nematode Infections (2010 Estimate)†^				0.03 (0.016–0.048)	[50]
Duration of Anemia (years)	1				Assumption
Population Attributable Fraction (PAF)*	0.28 (0.08)				[17, 18, 30, 51]
Probability of Anemia					
Children					
Low Estimate Study		0.129	0.133	0.143	[19]
Median Estimate Study		0.088	0.182	0.182	[18]
High Estimate Study		0.600	0.737	0.797	[17]
Adults					
Low Estimate Study		0.041	0.100	0.045	[18]
Median Estimate Study		0.486	0.610	0.720	[30]
High Estimate Study		0.697	0.740	0.803	[16]

*Mean (standard deviation), beta distribution

^ǂ^Only children aged 0–14 years with anemia due to moderate or high intensity infections were assumed to have cognitive impairment

†Estimate (95% uncertainty interval), triangular distribution

^Used for heavy intensity infections only

Health effects were measured in DALYs, which are the sum of the years of life lived with disability (YLD) and the years of life lost (YLL). However, since hookworm rarely causes death directly, our DALY calculation formula did not include YLLs and was therefore:

DALY=YLD=I*DW*L

where I is the number of incident cases, DW is the disability weight, and L is the average duration of the outcome (Table 1). YLDs accrued from anemia, heavy hookworm infection (>65 worms), and cognitive impairment (when included). The DW varied by the level of anemia depending on the scenario. As we aim to estimate the annual burden of hookworm, we assumed the duration of each outcome (i.e., hookworm-associated anemia and cognitive impairment) to be one year. Due to a lack of data on this outcome, we feel this is a conservative estimate given many people with hookworm would not receive treatment that would impact the duration of anemia or intensity of infection (e.g., annual MDA or iron-supplements); additionally, many studies looking at productivity losses evaluated a yearlong period[22].

Since a majority of infected individuals do not seek medical care for hookworm infection, our model did not include direct healthcare costs and instead focused on lost productivity (i.e., societal perspective). We assumed productivity losses accrue for all ages. To account for potential variability in assigning productivity reductions due to anemia, different scenarios explored the impact of using the following methodologies for estimating productivity losses for each person with hookworm-associated anemia (summarized in Table 2). The first method applied the DW for anemia to individual productivity for the duration of anemia (one year):

Productivity Losses=Per Capita Productivity*DW*L (1)

where the DW served as a proxy for the reduction in productivity and varied by the level of anemia, depending on the analysis scenario. Anemia levels were defined as: mild anemia 11–12.5g/dL for adults and 11g/dL for children, moderate anemia 8–10.9g/dL for all ages, and severe anemia <8g/dL for all ages.[23, 24] Productivity losses for individuals with heavy intensity infection and cognitive impairment were also considered using this method, using their respective DW estimates.

**Table 2:** Summary of methods used to estimate productivity losses.

Method	Formula*	Assumptions	Sources
1	Productivity Losses = Per Capita Productivity x Disability Weight x Duration	Disability weight serves as a proxy for reductions in productivity.	
2	Productivity Losses = Per Capita Productivity x (1-(Hemoglobin Level/Anemia Threshold)^1.5^)	Reductions in productivity due to anemia are determined from individual’s hemoglobin level relative to those without anemia. Elasticity of 1.5 estimates the change in work capacity or output divided by percent change in hemoglobin.	[25]
3	Productivity Losses = Per Capita Productivity x Loss of Productivity	Adapted from study on economic impact of anemia in a population and modified for an individual, assumes a 5% loss of productivity regardless of age.	[26, 27]

*Per capita productivity = a person’s median productivity contributions to society

The second method is based on the reduction in productivity due to anemia using methods described by Shastry and Weil.[25] This productivity reduction was calculated from an individual’s Hb level and determined the level of productivity in workers with anemia relative to those without anemia. The following formula calculated productivity losses using this method:

Productivity Losses=Per Capita Productivity*1-Hb LevelAnemia Threshold1.5 (2)

where 1.5 is the elasticity of productivity with respect to blood Hb among anemic workers.[25] Elasticity is the percent change in work capacity or output divided by percent change in Hb; thus, for every 1% increase in Hb levels, there will be a 1.5% increase in output. We used anemia thresholds of 12.5g/dL for adults and 11g/dL for children.[24]

The third method was adapted from a study on the economic impact of anemia in Peru[26] (which is based on a model by Ross and Horton[27]). This method calculates the cost due to productivity losses as:

Productivity Losses=Loss of Productivity*Wage Share*Gross Domestic Product per Capita*Prevalence

where loss of productivity is the loss of productivity for an adult with anemia; wage share is the ratio of employee compensation over the Gross Domestic Product (GDP) per capita; and prevalence is the prevalence of anemia in the population. As this formula results in the productivity loss per capita in the population due to anemia, we modified it to calculate the cost for an individual. Wage share and GDP per capita can be simplified to wage per capita; thus we used per capita productivity. Our modified formula is:

Productivity Losses=Loss of Productivity*Per Capita Productivity (3)

Previous work has estimated the loss of productivity in adults with anemia doing manual labor that is not highly demanding (e.g., technical staff, salespersons, etc.) to be 5% and those with highly demanding physical work (e.g., laborers or agricultural workers) to be 12–17%.[26] A systematic review estimated the percent of annual productivity loss for anemia due to soil-transmitted helminths ranged from 0.1% to 17%.[22] To remain conservative, we utilized 5% for all ages and did not stratify by work force type.

### Data Sources

Table 1 shows our overall model inputs while S1 Table provides country-specific values. The United Nations 2015 population estimates supplied the population size by age group.[28] Country-specific, age-stratified hookworm prevalence estimates consisted of a triangular prevalence distribution for each of the four modeled age groups.[29] We assumed the prevalence of hookworm has not changed substantially over time, given prevalence has only decreased 5% between 1990 and 2013.[6] The probability of anemia by hookworm infection intensity and by age group (children and adults) came from the literature.[16–19, 30] Depending on the scenario, gross national income (GNI) per capita or minimum wage served as a proxy for a person’s median productivity contributions to society (i.e., per capita productivity), but does not necessarily reflect that actual income of each person, which may be significantly less in impoverished areas. GNI per capita was obtained from the World Bank[31], supplemented with data from the United Nations[32] when not available from the World Bank. For the 14 countries for which GNI per capita was not available from either source, we utilized the average GNI from similar countries in the same region (defined by income classification when available or similar economies and industries). Minimum wage data was obtained from the US Department of State.[33] Again, for countries in which data was not available (31 countries), we utilized the average minimum wage from similar countries in the same region. All costs are presented in 2016 US dollars, converted using a 3% discount rate. DW values for hookworm and hookworm-associated health outcomes came from published estimates (Table 1).

### Simulation Scenarios

For each scenario, we ran 1,000 Monte Carlo simulations varying parameters throughout their ranges and report the median and 95% uncertainty interval (UI). Sensitivity analysis explored the impact of varying the following parameters: estimates for the probability of anemia for a given intensity of hookworm infection for children and adults (low, median, and high estimates from different studies in the literature, Table 1), the DW values (range: 2004 GBD estimates to the 2010 GBD estimates and additionally +/-10% of each of these two sets of estimates), the presence/absence of cognitive impairment, and annual wages (county-specific GNI per capita and minimum wage data). Additional scenarios evaluated the thresholds for low (<50 worms), moderate (50–105 worms), and heavy intensity (>105 worms) infection.[34]

## Results

### Health Effects as Measured by DALYs

**Fig 1:**
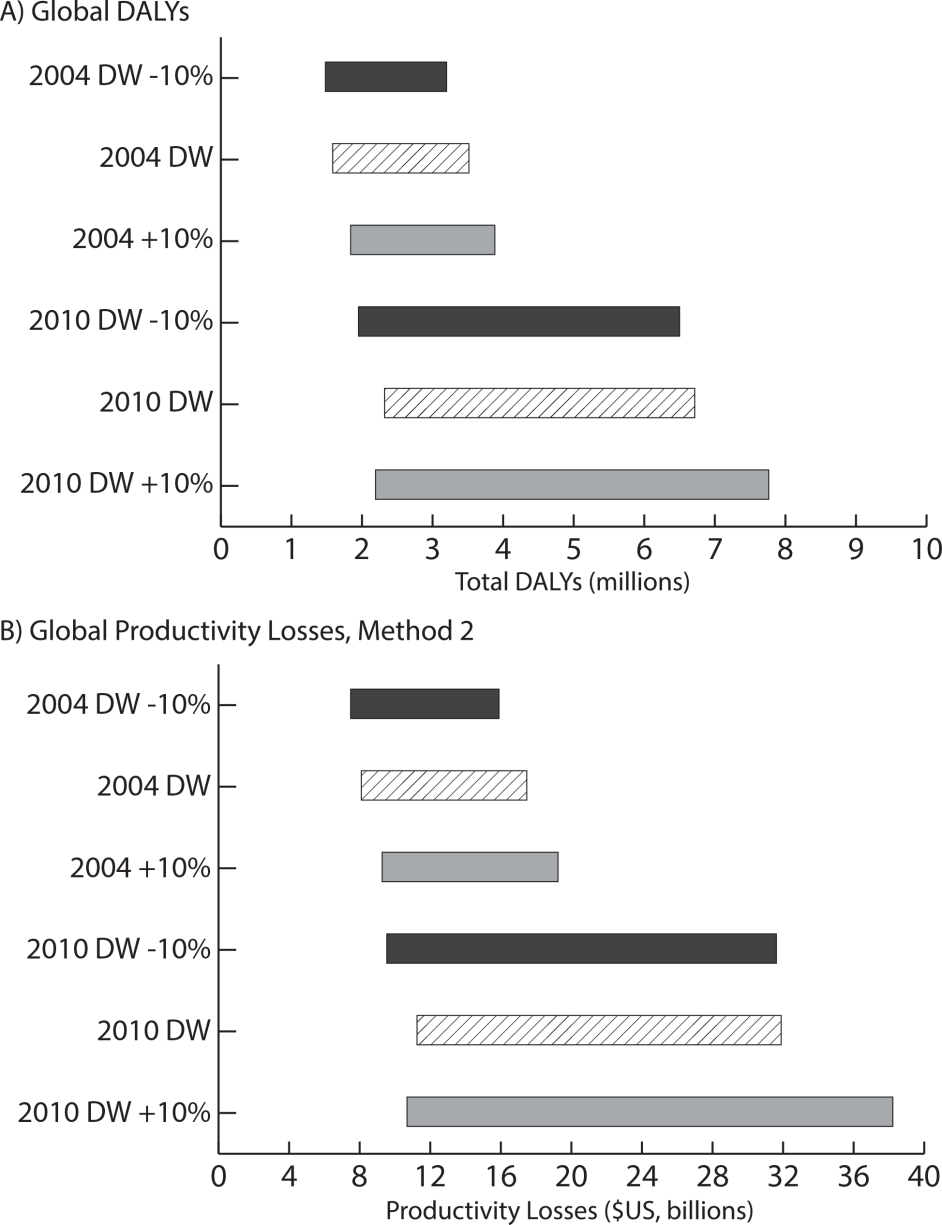
Impact of disability weight (DW) estimates on A) global DALYs, and B) global productivity losses across varying levels of anemia calculated using Method 1 (including cognitive impairment). The lower end of the range is the total burden assuming a low probability of anemia, while the upper end assumed a high probability of anemia. The 2004 DW and 2010 DW represent results with the published DW estimates and +/- 10% indicates adjustment to these values.

Table 3 presents the number of hookworm infections with consequent health outcomes (i.e., those with heavy intensity infection and hookworm-associated anemia,) and total DALYs accrued for each region and worldwide in 2016 (excluding cognitive impairment) using the 2004 DW estimates. The total number of DALYs varied with the probability of anemia given hookworm infection. Globally, 88,801,614 heavy hookworm infections and hookworm-associated anemia cases resulted in 2,126,280 DALYs (95% UI: 1,105,778–3,408,973), assuming the median likelihood of anemia. This translates to an average 0.0239 DALYs accrued (95% UI: 0.0150–0.0459 DALYs) annually per hookworm infection with consequent health outcomes. The Western Pacific region accrued the most DALYs. Including cognitive impairment increased the total DALYs accrued worldwide to 2,126,469 (95% UI: 1,105,873–3,409,135). Table 4 shows the DALYs accrued when using the 2010 DW estimates. This method resulted in an estimated 4,087,803 DALYs globally (95% UI: 386,827–17,054,291, median likelihood of anemia). Fig 1A shows how sensitive our DALY estimates are to the DW utilized (2004, 2010, and varying both by +/-10%) with the width of the bar indicating the range of total DALYs accrued across the varying likelihoods of anemia for each DW estimate evaluated. The 2010 DWs resulted in substantially more DALYs accrued than the 2004 DWs.

**Table 3:** Total annual hookworm-associated anemia cases and hookworm infections with consequent health outcomes, disability-adjusted life years (DALYs), and costs [median (95% uncertainty interval), in millions] due to hookworm infection by global region and worldwide in 2016 without cognitive impairment using the 2004 disability weight estimates and GNI per capita as a proxy for annual wages.

	Number with Hookworm-Associated Anemia	Number Hookworm Infections with Consequent Health Outcomes	Total DALYs^a^	Total Costs (Method 1, DW)^a^	Total Costs (Method 2, Hb Levels)	Total Costs (Method 3, Loss of Productivity)
**Low Estimate Study**						
Africa	14,786,448 (2,923,941–42,015,333)	15,073,893 (3,354,343–42,370,871)	357,582 (73,903–1,012,618)	835 (197–2,314)	5,936 (500–29,886)	1,687 (350–47,72)
Americas	2,897,418 (385,742–8,473,483)	2,897,418 (385,742–8,473,483)	69,538 (9,258–203,364)	622 (79–1,833)	4,359 (143–24,847)	1,295 (164–3,818)
Eastern Mediterranean	1,181,202 (166,087–3,484,810)	1,181,202 (166,087–3,484,810)	28,349 (3,986–83,635)	101 (13–298)	715 (22–4,101)	210 (27–621)
Europe	12,518 (713–38,742)	12,518 (713–38,742)	300 (17–930)	3 (0–8)	18 (0–102)	5 (0–16)
South-East Asia	15,389,380 (2,198,681–45,800,921)	15,389,380 (2,198,681–45,800,921)	369,345 (52,768–1,099,222)	737 (102–2,201)	5,320 (179–30,330)	1,534 (212–4,585)
Western Pacific	31,795,304 (3,737,079–100,206,191)	31,839,962 (3,899,100–100,273,205)	763,695 (90,110–2,406,237)	5,823 (658–18,432)	40,721 (1,024–235,929)	12,125 (1,362–38,395)
Worldwide	65,594,860 (9,444,920–199,553,212)	65,928,660 (9,919,954–199,793,382)	1,577,833 (230,769–4,792,622)	8,141 (1,062–25,148)	56,768 (1,864–318,404)	16,887 (2,137–52,344)
**Median Estimate Study**						
Africa	18,761,570 (9,646,619–29,951,265)	19,051,955 (9,990,010–30,143,441)	453,025 (234,065–721,702)	1,062 (571–1,660)	8,772 (749–26,966)	2,159 (1,135–3,384)
Americas	3,819,361 (1,983,431–6,291,344)	3,819,361 (1,983,432–6,291,344)	91,665 (47,602–150,992)	825 (422–1,370)	7,162 (170–22,065)	1,718 (879–2,855)
Eastern Mediterranean	1,548,628 (829,247–2,546,105)	1,548,628 (829,247–2,546,105)	37,167 (19,902–61,107)	133 (72–218)	1,141 (28–3,692)	276 (150–454)
Europe	17,591 (9,253–29,524)	17,591 (9,253–29,524)	422 (222–709)	4 (2–6)	30 (0–101)	7 (4–13)
South-East Asia	20,713,502 (10,609,194–34,340,345)	20,713,502 (10,609,194–34,340,345)	497,124 (254,621–824,168)	993 (517–1,652)	8,494 (209–27,338)	2,069 (1,078–3,441)
Western Pacific	43,279,170 (22,008,233–72,731,777)	43,391,267 (22,085,818–72,755,427)	1,039,728 (528,738–1,745,854)	7,899 (3,989–13,381)	65,597 (1,120–215,830)	16,455 (8,305–27,876)
Worldwide	88,446,791 (45,965,585–141832153)	88,801,614 (46,336,714–142,143,466)	2,126,280 (1,105,778–3,408,973)	10,976 (5,676–17,949)	90,763 (2,348–300,177)	22,805 (11,765–37,329)
**High Estimate Study**						
Africa	35,276,980 (18,402,627–56,010,460)	355,31,461 (18,815,101–56,204,962)	850,099 (444,902–1,348,344)	1,918 (1,018–3,019)	15,466 (2,479–40,757)	3,930 (2,070–6,226)
Americas	6,331,736 (3,249,533–10,297,000)	6,331,738 (3,249,534–10,297,001)	151,962 (77,989–247,128)	1,367 (691–2,248)	10,977 (1,101–30,452)	2,848 (1,440–46,83)
Eastern Mediterranean	2,646,728 (1,376,459–4,228,235)	2,646,728 (1,376,459–4,228,235)	63,521 (3,3035–101,478)	222 (114–350)	1,781 (168–4,951)	462 (237–730)
Europe	25,354 (12,885–41,666)	25,354 (12,885–41,666)	608 (309–1,000)	5 (3–8)	41 (0–133)	11 (5–18)
South-East Asia	34,669,373 (17,306,619–56,921,277)	34,669,373 (17,306,619–56,921,277)	832,065 (415,359–1,366,111)	1,641 (814–2,671)	13,165 (1,196–37,469)	3,419 (1,697–5,565)
Western Pacific	67,324,355 (34,639,136–121,283,469)	67,385,723 (34,671,441–121,287,445)	1,616,311 (831,409–2,910,886)	12,283 (6,265–22,206)	98,721 (7,582–306,239)	25,585 (13,049–46,258)
Worldwide	146,401,508 (76,158,881–245,420,243)	146,675,199 (76,471,898–245,631,435)	3,515,858 (1,830,282–5,894,024)	17,440 (8,958–29,837)	138,875 (12,690–412,953)	36,253 (18,593–62,067)

Note: Hb = hemoglobin; DW = disability weight

^a^Includes outcomes of anemia and heavy intensity infection

Global DALY estimates did not differ substantially when varying the infection intensity threshold. Threshold of 1 to 49, 50 to 104, and ≥105 worms resulted in 89,765,729 infections with consequent health outcomes globally. These infections generated 2,091,209 (95% UI: 1,105,275–3,462,856) DALYs using the 2004 DW estimates and 4,193,001 (95% UI: 395,922–16,499,971) DALYs using the 2010 DW estimates (median likelihood of anemia).

### Costs (Productivity Losses)

Table 3 also provides the productivity losses for hookworm-associated anemia in 2016 estimated from the three different calculation methods. Hookworm resulted in $11.0 billion (95% UI: $5.7–17.9 billion) in productivity losses (Method 1, median likelihood of anemia). Method 2, based on Hb levels, resulted in higher productivity losses overall, ranging from $56.8 to $138.9 billion annually, varying with the likelihood of anemia. Method 3 also resulted in higher productivity losses, ranging from $16.9 to 36.3 billion, depending on the likelihood of anemia. As Method 1 is more conservative (i.e., results in the lower economic burden) and more inclusive in health outcomes (i.e., includes infection, anemia, and cognitive impairment), the rest of the results utilized this method.

Including the cognitive impairment outcome had little effect on total costs. Inclusion increased global costs by approximately $650,000, $1.2 million, and $4.3 million annually for low, median, and high likelihoods of anemia, respectively. None of these increases resulted in significantly different global cost estimates. Differences were primarily seen in the African region, where more medium and heavy intensity infections occur.

Globally, total productivity losses were sensitive to the DWs utilized when calculating costs via Method 1 (Fig 1B). Although there was some overlap between cost estimates, substantial variability arose when using the 2010 DW values, especially across the different likelihoods of anemia. A 10% increase in the 2010 DW values with a high likelihood of anemia resulted in productivity losses of $38.2 billion annually worldwide. Using the 2010 DW estimates substantially increased the economic burden of hookworm (Table 4). Assuming a median likelihood of anemia, hookworm cost $20.9 billion (95% UI: $1.8–89.1 billion) worldwide. The low estimate for anemia yielded $11.2 billion (95% UI: $1.6–83.1 billion) in total costs.

**Table 4:** Total annual hookworm-associated anemia cases and hookworm infections with consequent health outcomes, disability-adjusted life years (DALYs), and costs [median (95% uncertainty interval), in millions] due to hookworm infection by global region and worldwide in 2016 using the 2010 disability weight estimates and GNI per capita as a proxy for annual wages.

	Number with Hookworm-Associated Anemia	Number Hookworm Infections with Consequent Health Outcomes	Total DALYs^a^	Total Costs (Method 1, DW)^a^
**Low Estimate Study**				
Africa	14,397,791 (3,253,941–39,263,583)	14,508,448 (3,714,312–37,616,985)	594,303 (136,519–3,475,798)	1,442 (414–7,748)
Americas	2,768,102 (464,302–8,217,664)	2,782,924 (437,366–8,055,027)	96,333 (12,925–715,775)	849 (112–6,493)
Eastern Mediterranean	1,135,944 (199,126–3,286,609)	1,121,787 (198,913–3,260,959)	40,326 (6,215–291,279)	138 (20–1,019)
Europe	12,414 (1,034–37,289)	12,043 (1,048–37,555)	355 (9–3421)	3 (0–27)
South-East Asia	15,289,602 (2,547,997–44,979,096)	14,900,576 (2,546,556–42,332,600)	535,843 (75,011–3,754,327)	1,065 (144–7,493)
Western Pacific	30,736,718 (4,284,618–92,737,620)	30,596,812 (4,528,414–92,448,906)	1,028,365 (125,562–7,991,354)	7,745 (885–60,568)
Worldwide	64,573,661 (11,342,956–181,783,349)	64,683,374 (11,240,779–185,834,213)	2,308,459 (355,948–15,962,498)	11,225 (1,634–83,120)
**Median Estimate Study**				
Africa	18,817,718 (9,531,678–295,20,877)	18,722,900 (10,096,669–30,448,580)	892,077 (133,495–3,304,041)	2,125 (410–7,779)
Americas	3,800,168 (1,945,997–6,158,672)	3,687,266 (1,910,239–6,258,202)	175,719 (14,158–725,074)	1,573 (125–6,613)
Eastern Mediterranean	1,551,203 (757,976–2,477,911)	1,520,231 (796,265–2,564,340)	71,290 (6,520–290,076)	256 (21–1,040)
Europe	17,420 (8,232–27,665)	17,388 (8,846–29,932)	786 (35–3,360)	6 (0–28)
South-East Asia	20,627,972 (10,326,213–33,725,352)	20,510,540 (10,602,277–34,437,672)	970,366 (81,755–3,834,583)	1,955 (158–7,678)
Western Pacific	42,528,706 (20,207,813–71,510,519)	42,061,785 (21,720,559–75,229,080)	1,917,887 (142,184–8,497,810)	14,632 (1,024–64,791)
Worldwide	88,622,934 (43,380,184–139,089,685)	86,972,676 (46,632,977–147,115,026)	4,087,803 (386,827–17,054,291)	20,877 (1,779–89,060)
**High Estimate Study**				
Africa	35,255,094 (18,833,338–56,829,822)	35,680,358 (18,873,329–55,115,211)	1,728,724 (480,023–5,892,707)	3,900 (1,174–13,393)
Americas	6,349,667 (3,445,570–10,629,166)	6,329,855 (3,131,696–10,058,177)	283,015 (54,181–1,101,617)	2,503 (471–9,885)
Eastern Mediterranean	2,623,645 (1,389,973–4,319,973)	2,647,969 (1,391,489–4,217,500)	120,864 (24,791–465,864)	419 (80–1,604)
Europe	25,053 (12,842–43,202)	25,223 (12,738–41,219)	1,068 (58–4,933)	9 (0–41)
South-East Asia	3,517,1442 (18,608,681–57,235,451)	34,987,826 (17,894,163–56,186,335)	1,573,595 (295,314–5,911,227)	3,112 (571–11,854)
Western Pacific	68,095,564 (33,944,724–118,129,017)	68,980,957 (34,789,309–112,180,735)	2,903,578 (475,555–13,203,711)	22,116 (3,491–101,491)
Worldwide	148,656,202 (76,567,520–242,268,723)	148,993,910 (77,795,887–236,527,374)	6,703,380 (1,347,676–27,054,100)	31,866 (5,670–140,376)

Note: Hb = hemoglobin; DW = disability weight

^a^Includes outcomes of anemia and heavy intensity infection

**Fig 2:**
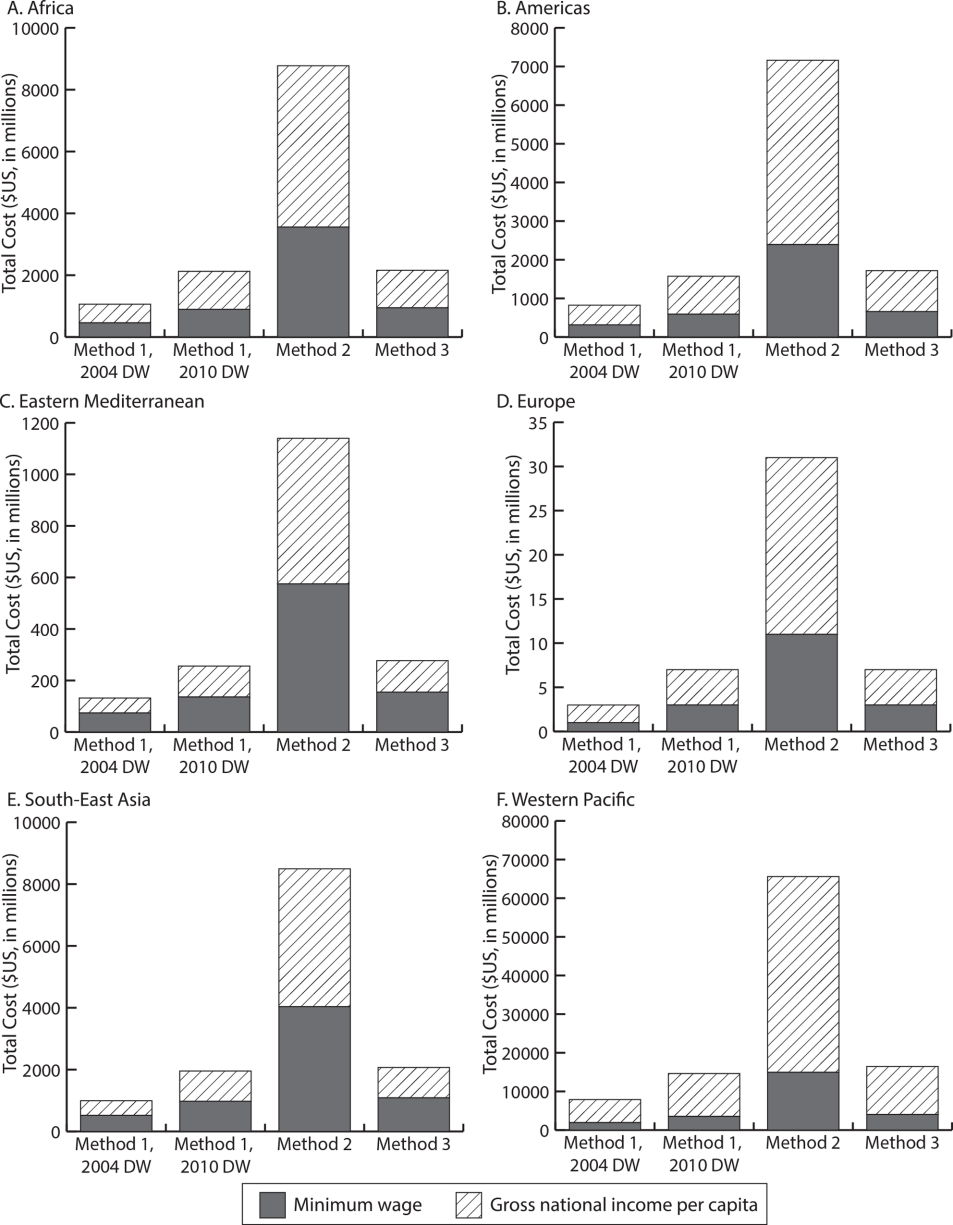
Hookworm-associated productivity losses in 2016 by annual wage proxy used for A) Africa, B) the Americas, C) Eastern Mediterranean, D) Europe, E) South-East Asia, and F) Western Pacific regions.

Using minimum wage data as a proxy for per capita productivity, hookworm results in productivity losses totaling $2.5 to $5.4 billion using Method 1 and 2004 DW estimates, $3.4 to $10.5 billion using Method 1 and 2010 DW estimates, $17.5 to $43.9 billion using Method 2, and $5.1 to $11.3 billion using Method 3, varying with the likelihood of anemia (low to high). Fig 2 shows how the burden estimates differ by region, annual wage, and productivity loss method used. In most regions, using the GNI per capita resulted in a burden 1.8 to 2.7 times higher (depending on the region) than when using the minimum wage, regardless of the calculation method, with the exception the Western Pacific, where estimates using GNI per capita were 4.0 fold higher than using minimum wage.

The cost per hookworm infection with consequent health outcomes (i.e., heavy intensity infection, hookworm-associated anemia, and cognitive impairment) is an estimated $56 (95% UI: $52–59) in Africa, $215 (95% UI: $203–227) in the Americas, $86 (95% UI: $80–94) in the Eastern Mediterranean, $202 (95% UI: $184–216) in Europe, $48 (95% UI: $45–51) in South East Asia, and $183 (95% UI: $178–187) in the Western Pacific (Method 1, median likelihood of anemia, 2004 DW estimates, GNI per capita). Using the 2010 DW estimates, these increased to $120 (95% UI: $24–415) in Africa, $447 (95% UI: $40–1,693) in the Americas, $176 (95% UI: $17–672) in the Eastern Mediterranean, $406 (95% UI: $20–1,595) in Europe, $99 (95% UI: $9–370) in South East Asia, and $375 (95% UI: $30–1,414) in the Western Pacific. Using minimum wage as a proxy for per capita productivity, the cost per hookworm infection is an estimated $24 (95% UI: $23–25) in Africa, $82 (95% UI: $77–89) in the Americas, $47 (95% UI: $45–50) in the Eastern Mediterranean, $80 (95% UI: $71–87) in Europe, $25 (95% UI: $24–26) in South East Asia, and $45 (95% UI: $44–45) in the Western Pacific (using 2004 DW estimates). There is considerable variation in different countries due to per capita productivity proxy assumed (GNI per capita vs. minimum wage), DW estimates used (2004 vs. 2010), and variations in the 2010 DW estimates.

A closer look at individual countries shows that China’s 35,917,360 hookworm infections with consequent health outcomes result in $6.7 billion in productivity losses using GNI per capita, $1.7 billion in productivity losses using minimum wage, and 862,017 DALYs annually. In India, 11,781,041 hookworm-associated health outcomes cost $471 million (GNI per capita; $258 million using minimum wage) with 282,75 DALYs lost. Brazil’s estimated 1,355,874 infections with consequent health outcomes cost $398 million and $80 million in productivity losses using GNI per capita and minimum wage, respectively (and 32,541 DALYs) annually. In Africa, Nigeria harbors the largest hookworm burden with 3,738,750 infections with consequent health outcomes costing $283 million using GNI per capita and $99 million using minimum wage, and accruing 89,730 DALYs.

Again, results were robust to changes in the infection intensity threshold. Assuming a median likelihood of anemia, 2004 DW estimates, and GNI per capita, 89,765,729 hookworm infections that accrue health outcomes globally resulted in productivity losses totaling $11.1 billion (95% UI: $5.5–18.5 billion) utilizing Method 1, $87.9 billion (95% UI: $1.7–302.3 billion) utilizing Method 2, and $23.1 (95% UI: $11.4–38.4 billion) utilizing Method 3. Using minimum wage, the global burden calculated using Method 1 was $3.3 billion (2004 DW estimates) and $6.5 billion (2010 DW estimates); using Method 2 and 3, productivity losses totaled $25.1 billion and $6.8 billion, respectively.

## Discussion

Even though hookworm is classified as a neglected tropical disease, its economic and health burden (ranging from $7.5 billion to $138.9 billion using GNI per capita and ranging from $2.4 billion to $43.9 billion using minimum wage) exceeded published estimates for a number of diseases that have received comparatively more attention than hookworm. For example, rotavirus cost an estimated $423 million ($262–590 million) to society in low- and middle-income countries in the absence of vaccination (2007 values)[35], and $2 billion annually globally (2007 values).[36] Annual estimates for tuberculosis suggest $12 billion in productivity losses alone (assuming a 30% reduction in productivity and loss of 15 income years per death).[37] Dengue cost an estimated $12.3 billion worldwide in 2010.[38] Although the general methodologies of these studies are similar (identifying the number of cases and associated unit costs), caution should be taken when making direct comparisons as there is variation in specific costs included. Our study shows how the cumulative economic impact of a subacute chronic disease like hookworm can eventually exceed the impact of diseases that have higher mortality and more salient health effects.

Quantifying the economic burden of a disease can help aid decision makers, such as funding agencies and public health bodies, make informed decisions about where to best allocate limited resources and gauge investments and potential returns for intervention and control measures. Showing the burden can also tell investors and manufacturers, how much can be invested in prevention and control strategies, and provide motivation for research and development of new strategies (e.g., vaccines). These investments can not only improve the health of those with hookworm, but support the economic growth of affected regions as they become more economically productive.

Our study also identified potential targets for future studies and data collection. These are parameters whose values substantially influence the burden estimates. Our results were sensitive to the disability weights used, the calculation method used to determine productivity losses due to anemia, and the probability of hookworm-associated anemia (which likely varies across regions). Variability in these parameters, along with differences in outcomes included and methodologies to attribute Hb levels, account for differences between our DALY estimates and those published by the GBD. The revised GBD study for 2010 reports the loss of 3,230,800 DALYs annually due to hookworm among all ages in 21 regions.[29] This estimate included disability weights for additional hookworm outcomes (wasting and mild abdominopelvic problems) and stratified by level of anemia, but did not include cognitive impairment.

Another finding is that certain large countries that are transitioning to higher income countries such Brazil and China, still face considerable hookworm burden. These countries are investing heavily in infrastructure and various industries but it is not clear how much they may be spending on controlling hookworm.

All models are simplifications of real life[39] and therefore cannot represent every possible hookworm event or outcome. Other diseases occurring in the same geographic area cause anemia and cognitive impairment (e.g., malaria, schistosomiasis, and meningitis), which may make it difficult to attribute the true underlying cause and infected individuals may not seek care or be appropriately diagnosed.[40] Data used in our model came from a variety of sources and studies of variable quality; therefore our results may change as more data becomes available. For example, hookworm prevalence data is limited; it tends to be extrapolated from localized cross-sectional surveys, many of which are done in high prevalence areas with differing data collection methods. It should be noted that there is some uncertainty in infection intensity thresholds, as they are based on limited data. Additionally, these thresholds would ideally vary for different hookworm species. For example, *Ancylostoma duodenale* causes greater blood loss than *Necator americanus*[41] so it would require fewer *A*. *duodenale* worms to cause the same blood loss as an *N*. *americanus* infection. However, as *A*. *duodenale* causes less than 15% of the global hookworm burden[42–44], our model did not differentiate between hookworm species. Additionally, while a few studies have estimated the impact of anemia on worker productivity[22], the true impact is not well understood or quantified. Many variations between studies make them difficult to compare (e.g., geographically focal, involve very specific populations, productivity loss definitions vary, studies are older, measure of productivity and length of time vary) and generalizability is a concern. Other difficulties to measuring productivity losses include underlying and/or concomitant health conditions that may also affect productivity and a lack of studies designed to measure the impact of hookworm infection directly on productivity (compared to hookworm-associated outcomes).

Our estimates may in fact be quite conservative. We did not consider other possible outcomes of hookworm (e.g., wasting and impact on physical growth) as they are difficult to quantify. When using the 2010 DW estimates, the weight for symptomatic intestinal nematode infections was applied only to those with heavy intensity infection. We also considered disability from cognitive impairment (when utilizing the 2004 disability weight estimates) and while chronic hookworm infection can lead to cognitive impairment[1, 17, 41, 45, 46], there is some controversy and uncertainty regarding the amount of cognitive impairment caused by hookworm. In fact, cognitive impairment was removed from the 2010 GBD estimates. Additionally, we did not include the potential impact of long-term cognitive impairment through adulthood. Some studies show that chronic hookworm in childhood reduces future earnings in adults[47] and that school-based deworming can have a differential impact on future wages[48]. However, there is little data on the number of adults with long-term hookworm-associated cognitive impairment, making it difficult to differentiate between cognitive impairment in active vs. past infections, especially in endemic areas. Thus to remain conservative, we excluded this outcome. The cost calculation using Method 3 is likely an underestimate as we did not account for those who perform heavy manual labor, and their reduction in productivity due to anemia may be greater. Also, estimating at the population level may have underestimated the true burden of disease (i.e., infection intensity) that may result in areas with a higher prevalence (i.e., our M was calculated at a country level and thus may underestimate the number of medium and heavy intensity infection in high prevalence areas). This in turn would underestimate hookworm’s clinical outcomes. Countries for which there was no hookworm prevalence data and where conditions are not suitable for hookworm, were not included in this study. However, it is possible that there may be a small burden of hookworm in these countries.

Human hookworm infection results in a substantial economic and health burden globally, which surpasses published estimates for other diseases. Adults bear most of the costs and while the total economic burden is highest in the Western Pacific region, certain large countries that are transitioning into the higher income bracket such Brazil and China, still face considerable hookworm burden. Interventions (such as a vaccine or community-wide treatment) to reduce the disease burden of hookworm among all age groups could have substantial impacts on this burden.

## Supporting Information

S1 Table County-specific input parameters. (DOCX)
