# Supplementary material for: The Global Economic and Health Burden of Human Hookworm Infection
Source: PLoS Negl Trop Dis. 2016 Sep 8;10(9):e0004922. doi: 10.1371/journal.pntd.0004922 (PMC5015833; doi:10.1371/journal.pntd.0004922)
Supplement: S1 Table — (DOCX) [file pntd.0004922.s001.docx]

**S1 Table. County-specific input parameters**

|  | **Population Counts** | | | | **Gross National Income per Capita (2016 $US)** | **Minimum Wage (2016 $US)** |
| --- | --- | --- | --- | --- | --- | --- |
| **Country** | **0 to 4 years old** | **5 to 9 years old** | **10 to 14 years old** | **15 years and older** |  |  |
| **Africa** | | | | | |  |
| Algeria | 4,589,813 | 3,817,053 | 2,913,447 | 28,346,206 | 5,814 | 2,310 |
| Angola | 4,717,892 | 3,934,657 | 3,270,532 | 13,098,893 | 5,145 | 1,916 |
| Benin | 1,707,733 | 1,523,648 | 1,355,381 | 6,293,067 | 859 | 878 |
| Botswana | 266,262 | 236,661 | 221,029 | 1,538,533 | 7,681 | 737 |
| Burkina Faso | 3,143,912 | 2,756,975 | 2,350,195 | 9,854,488 | 753 | 692 |
| Burundi | 2,061,897 | 1,651,675 | 1,294,732 | 6,170,617 | 286 | 237 |
| Cameroon | 3,737,925 | 3,294,047 | 2,894,600 | 13,417,607 | 1,443 | 779 |
| Cape Verde | 53,589 | 51,323 | 49,420 | 366,170 | 3,660 | 1,347 |
| Central African Republic | 708,095 | 620,821 | 584,769 | 2,986,589 | 350 | 185 |
| Chad | 2,632,347 | 2,200,281 | 1,866,101 | 7,338,743 | 1,040 | 1,285 |
| Comoros | 119,289 | 105,412 | 92,847 | 470,926 | 870 | 1,545 |
| Congo, Rep. | 759,042 | 656,315 | 554,323 | 2,650,650 | 2,875 | 1,928 |
| Cote d'Ivoire | 3,667,067 | 3,156,716 | 2,818,548 | 13,059,225 | 1,549 | 531 |
| Democratic Republic of the Congo | 13,875,564 | 11,739,774 | 9,921,938 | 41,729,538 | 403 | 688 |
| Equatorial Guinea | 128,218 | 109,835 | 93,725 | 513,282 | 13,410 | 2,766 |
| Eritrea | 814,756 | 767,862 | 654,103 | 2,991,070 | 721 | 297 |
| Ethiopia | 14,601,687 | 13,612,096 | 12,973,970 | 58,202,997 | 583 | 247 |
| Gabon | 239,080 | 212,092 | 189,376 | 1,084,744 | 10,026 | 3,214 |
| Gambia | 366,225 | 301,178 | 252,091 | 1,071,430 | 467 | 470 |
| Ghana | 4,055,985 | 3,494,337 | 3,089,414 | 16,770,157 | 1,697 | 699 |
| Guinea | 2,045,642 | 1,767,715 | 1,549,328 | 7,245,905 | 499 | 813 |
| Guinea Bissau | 288,795 | 246,715 | 216,844 | 1,091,971 | 583 | 408 |
| Kenya | 7,166,489 | 6,486,995 | 5,645,766 | 26,751,052 | 1,369 | 816 |
| Lesotho | 278,395 | 249,221 | 242,543 | 1,364,863 | 1,422 | 1,051 |
| Liberia | 701,125 | 637,288 | 566,799 | 2,598,226 | 393 | 364 |
| Madagascar | 3,770,130 | 3,328,039 | 3,010,251 | 14,126,970 | 467 | 507 |
| Malawi | 2,953,595 | 2,613,405 | 2,206,879 | 9,441,353 | 265 | 451 |
| Mali | 3,271,256 | 2,802,532 | 2,290,675 | 9,235,231 | 700 | 618 |
| Mauritania | 601,082 | 542,441 | 484,170 | 2,439,871 | 1,347 | 1,125 |
| Mauritius | 70,624 | 80,641 | 94,747 | 1,027,200 | 10,301 | 1,178 |
| Mayotte*ǂ | 35,418 | 34,951 | 29,667 | 139,979 | 9,331 | 2,121 |
| Mozambique | 4,816,063 | 4,203,524 | 3,655,369 | 15,302,907 | 658 | 890 |
| Namibia | 338,241 | 292,269 | 271,638 | 1,556,682 | 6,026 | 1,051 |
| Niger | 4,144,755 | 3,280,389 | 2,617,062 | 9,856,914 | 446 | 643 |
| Nigeria | 31,109,162 | 26,487,539 | 22,552,424 | 102,052,837 | 3,151 | 1,112 |
| Reunion*ǂ | 65,087 | 68,106 | 70,289 | 657,672 | 9,331 | 2,121 |
| Rwanda | 1,694,881 | 1,644,841 | 1,426,280 | 6,843,664 | 743 | 177 |
| Sao Tome and Principe | 29,576 | 27,031 | 24,532 | 109,205 | 1,772 | 600 |
| Senegal | 2,601,312 | 2,171,094 | 1,848,481 | 8,508,386 | 1,103 | 771 |
| Seychelles | 8,379 | 7,699 | 6,540 | 73,853 | 14,842 | 3,771 |
| Sierra Leone | 1,004,248 | 921,109 | 808,401 | 3,719,426 | 753 | 1,162 |
| South Africa | 5,370,121 | 5,511,849 | 5,050,605 | 38,557,831 | 7,214 | 2,335 |
| Swaziland | 173,392 | 159,704 | 147,651 | 806,223 | 2,864 | 420 |
| Tanzania | 9,398,450 | 8,019,385 | 6,750,096 | 29,302,489 | 987 | 247 |
| Togo | 1,160,024 | 1,032,923 | 892,443 | 4,219,188 | 605 | 754 |
| Uganda | 7,277,920 | 6,227,286 | 5,265,723 | 20,261,454 | 721 | 51 |
| Western Sahara* | 50,497 | 50,004 | 46,851 | 425,188 | 2,628 | 2,707 |
| Zambia | 2,850,804 | 2,487,553 | 2,105,260 | 8,768,150 | 1,782 | 1,067 |
| Zimbabwe | 2,504,682 | 2,151,142 | 1,834,974 | 9,111,953 | 881 | 3,041 |
| **Americas** | | | | | |  |
| Anguillaǂ | 1,174 | 1,152 | 1,148 | 12,944 | 21,562 | 8,523 |
| Antigua and Barbuda | 7,280 | 7,300 | 7,620 | 69,618 | 14,174 | 6,491 |
| Argentina | 3,718,140 | 3,638,344 | 3,582,019 | 32,478,252 | 15,022 | 7,119 |
| Aruba | 5,242 | 6,641 | 7,167 | 84,839 | 25,842 | 11,297 |
| Bahamas | 29,112 | 27,137 | 24,929 | 306,841 | 22,258 | 11,248 |
| Barbadosǂ | 17,360 | 18,578 | 19,103 | 229,174 | 15,645 | 8,523 |
| Belize | 39,453 | 37,880 | 39,271 | 242,683 | 5,170 | 3,535 |
| Bermudaǂ | 3,749 | 4,028 | 4,203 | 53,201 | 118,514 | 8,523 |
| Bolivia | 1,185,563 | 1,160,118 | 1,133,975 | 7,245,049 | 3,087 | 2,966 |
| Brazil | 15,032,203 | 15,407,519 | 17,422,671 | 159,985,135 | 12,232 | 2,472 |
| British Virgin Islandsǂ | 2,393 | 2,347 | 2,339 | 26,374 | 32,305 | 8,523 |
| Cayman Islandsǂ | 4,232 | 4,152 | 4,138 | 46,650 | 58,843 | 8,523 |
| Chile | 1,170,411 | 1,199,567 | 1,246,022 | 14,332,141 | 15,818 | 3,214 |
| Colombia | 3,738,278 | 3,905,293 | 4,069,625 | 36,515,508 | 8,455 | 2,670 |
| Costa Rica | 350,327 | 360,487 | 361,753 | 3,735,283 | 10,736 | 3,955 |
| Cuba | 587,755 | 613,845 | 655,506 | 9,532,456 | 7,522 | 111 |
| Dominica | 5,174 | 5,076 | 5,059 | 57,032 | 7,501 | 3,171 |
| Dominican Republic | 1,062,223 | 1,059,300 | 1,032,661 | 7,374,207 | 6,397 | 1,887 |
| Ecuador | 1,610,333 | 1,567,986 | 1,506,658 | 11,459,386 | 6,440 | 4,375 |
| El Salvador | 519,884 | 545,432 | 590,614 | 4,470,653 | 4,191 | 2,568 |
| Falkland Islands*ǂ | 163 | 169 | 162 | 2,409 | 17,679 | 6,802 |
| French Guiana*ǂ | 32,630 | 30,963 | 27,563 | 177,450 | 12,131 | 6,304 |
| Grenada | 9,935 | 9,459 | 8,897 | 78,534 | 8,328 | 3,578 |
| Guadeloupe*ǂ | 31,223 | 35,534 | 36,168 | 365,525 | 17,679 | 6,802 |
| Guatemala | 2,089,433 | 1,972,262 | 1,923,535 | 10,357,667 | 3,618 | 3,534 |
| Guyana | 67,137 | 64,159 | 89,678 | 546,111 | 4,424 | 2,163 |
| Haiti | 1,237,543 | 1,212,462 | 1,164,056 | 7,097,006 | 870 | 1,059 |
| Honduras | 815,929 | 857,171 | 891,765 | 5,510,195 | 2,419 | 3,028 |
| Jamaica | 203,732 | 217,862 | 237,028 | 2,134,713 | 5,496 | 2,517 |
| Martinique*ǂ | 20,365 | 22,233 | 25,583 | 328,244 | 17,679 | 6,802 |
| Mexico | 11,616,909 | 11,606,866 | 11,839,884 | 91,953,565 | 10,460 | 1,594 |
| Montserrat* | 375 | 368 | 367 | 4,132 | 11,910 | 8,523 |
| Netherlands Antilles (Curacao)ǂ | 10,540 | 9,332 | 9,959 | 127,372 | 21,591 | 8,523 |
| Nicaragua | 605,548 | 617,263 | 603,884 | 4,255,337 | 1,984 | 1,891 |
| Panama | 368,407 | 356,020 | 342,907 | 2,861,807 | 11,808 | 2,613 |
| Paraguay | 673,789 | 669,634 | 657,199 | 4,638,501 | 4,647 | 2,348 |
| Peru | 3,020,032 | 2,903,953 | 2,828,731 | 22,623,954 | 6,758 | 2,781 |
| Puerto Ricoǂ | 220,740 | 227,953 | 246,578 | 2,987,967 | 20,773 | 8,523 |
| St. Kitts and Nevis | 3,930 | 3,855 | 3,842 | 43,317 | 15,372 | 7,134 |
| St. Luciaǂ | 13,771 | 14,053 | 14,958 | 142,217 | 7,511 | 4,881 |
| St. Vincent and the Grenadines | 8,512 | 9,091 | 9,247 | 82,612 | 6,960 | 5,479 |
| Suriname | 48,185 | 48,576 | 48,658 | 397,556 | 10,487 | 1,854 |
| Trinidad and Tobago | 96,103 | 96,415 | 90,107 | 1,077,463 | 16,151 | 5,056 |
| Turks and Caicos Islandsǂ | 2,413 | 2,367 | 2,359 | 26,600 | 25,513 | 8,523 |
| Uruguay | 240,761 | 244,603 | 249,503 | 2,696,688 | 17,346 | 4,079 |
| Venezuela | 2,959,946 | 2,922,847 | 2,860,262 | 22,365,028 | 13,675 | 14,585 |
| **Eastern Mediterranean** |  |  |  |  |  |  |
| Afghanistan | 4,950,250 | 4,903,273 | 4,471,809 | 18,201,230 | 711 | 1,174 |
| Bahrain | 108,728 | 99,858 | 87,175 | 1,081,476 | 23,468 | 10,012 |
| Djibouti | 102,054 | 96,033 | 92,558 | 597,216 | 1,946 | 2,447 |
| Egypt, Arab Repub. | 12,116,075 | 9,579,287 | 8,648,975 | 61,163,747 | 3,236 | 1,916 |
| Iran, Islamic Repub. | 6,855,319 | 6,395,371 | 5,426,591 | 60,431,991 | 6,907 | 2,942 |
| Iraq | 5,727,445 | 4,974,419 | 4,225,448 | 21,496,083 | 6,705 | 1,916 |
| Jordan | 980,405 | 896,019 | 821,571 | 4,896,552 | 5,474 | 3,593 |
| Kuwait | 347,875 | 298,135 | 222,870 | 3,023,235 | 60,984 | 1,854 |
| Lebanon | 461,133 | 438,539 | 504,099 | 4,446,972 | 10,397 | 6,078 |
| Libya | 649,002 | 649,582 | 574,453 | 4,405,401 | 8,392 | 4,561 |
| Morocco | 3,420,734 | 3,085,731 | 2,851,802 | 25,019,244 | 3,161 | 2,696 |
| Oman | 385,293 | 306,306 | 229,896 | 3,569,046 | 22,578 | 10,444 |
| Pakistan | 24,663,726 | 21,989,726 | 19,488,840 | 122,782,582 | 1,496 | 1,458 |
| Qatarǂ | 132,380 | 111,966 | 102,616 | 1,888,393 | 100,160 | 10,228 |
| Saudi Arabia | 3,161,462 | 3,100,108 | 2,753,303 | 22,525,499 | 28,369 | 9,888 |
| Somalia | 1,971,023 | 1,669,722 | 1,397,379 | 5,748,980 | 140 | 1,146 |
| Sudan | 5,952,169 | 5,409,518 | 4,934,753 | 23,938,442 | 1,814 | 791 |
| Syrian Arab Republic | 2,191,604 | 2,342,039 | 2,334,955 | 11,633,815 | 1,719 | 618 |
| Tunisia | 982,135 | 855,724 | 790,604 | 8,625,091 | 4,604 | 1,755 |
| United Arab Emiratesǂ | 491,344 | 431,840 | 353,065 | 7,880,714 | 47,953 | 10,228 |
| Yemen | 3,924,578 | 3,616,043 | 3,263,049 | 16,028,545 | 1,486 | 1,211 |
| **Europe** |  |  |  |  |  |  |
| Armenia | 207,244 | 188,829 | 158,700 | 2,462,939 | 4,010 | 1,298 |
| Azerbaijan | 930,423 | 618,622 | 588,883 | 7,616,040 | 8,052 | 801 |
| Georgia | 274,768 | 228,517 | 189,781 | 3,306,746 | 3,947 | 593 |
| Israel | 831,851 | 740,534 | 672,115 | 5,819,536 | 37,121 | 13,732 |
| Kazakhstanǂ | 1,948,448 | 1,618,984 | 1,141,570 | 12,916,224 | 12,381 | 5,488 |
| Kyrgyz Republic | 780,368 | 601,890 | 482,988 | 4,074,716 | 1,326 | 103 |
| Tajikistan | 1,175,607 | 941,052 | 838,211 | 5,526,985 | 1,146 | 618 |
| Turkey | 6,820,869 | 6,670,184 | 6,702,865 | 58,471,912 | 11,500 | 5,031 |
| Turkmenistan | 527,727 | 497,656 | 491,235 | 3,856,884 | 8,508 | 1,891 |
| Uzbekistan | 3,194,935 | 2,838,397 | 2,492,266 | 21,367,890 | 2,217 | 607 |
| **South-East Asia** | | | | | |  |
| Bangladesh | 15,331,344 | 15,667,941 | 16,409,061 | 113,587,296 | 1,146 | 816 |
| Bhutan | 65,934 | 71,519 | 70,707 | 566,670 | 2,536 | 669 |
| India | 123,711,487 | 126,965,226 | 126,750,182 | 933,623,632 | 1,666 | 902 |
| Indonesia | 24,863,562 | 22,923,744 | 23,538,263 | 186,238,246 | 3,851 | 1,026 |
| Korea, Dem. Rep. | 1,747,397 | 1,709,621 | 1,867,715 | 19,830,584 | 680 | 393 |
| Maldives | 37,364 | 32,750 | 29,814 | 263,729 | 7,607 | 2,546 |
| Myanmar | 4,564,913 | 4,988,273 | 5,295,804 | 39,048,164 | 1,347 | 545 |
| Nepal | 2,807,130 | 3,139,169 | 3,367,447 | 19,199,954 | 774 | 987 |
| Sri Lanka | 1,643,013 | 1,748,853 | 1,698,606 | 15,624,538 | 3,607 | 840 |
| Thailand | 3,798,996 | 4,035,979 | 4,201,152 | 55,923,232 | 5,697 | 3,120 |
| Timor-Leste | 203,732 | 149,174 | 149,565 | 682,294 | 3,310 | 1,421 |
| **Western Pacific** | | | | | |  |
| Cambodia | 1,771,553 | 1,660,542 | 1,490,876 | 10,654,928 | 1,082 | 1,680 |
| Chinaǂ | 83,185,944 | 78,637,404 | 75,291,908 | 1,138,933,687 | 7,829 | 1,917 |
| Cook Islandsǂ | 948 | 958 | 988 | 6,944 | 17,486 | 5,608 |
| Fiji | 88,080 | 88,025 | 80,339 | 635,701 | 4,816 | 2,288 |
| French Polynesia | 22,541 | 17,320 | 22,746 | 220,157 | 25,310 | 5,608 |
| Guam*ǂ | 14,247 | 14,198 | 14,819 | 126,621 | 50,517 | 5,608 |
| Kiribati | 14,757 | 13,727 | 10,804 | 73,135 | 2,281 | 2,656 |
| Korea, Repub.ǂ | 2,287,286 | 2,272,439 | 2,477,685 | 43,256,029 | 28,740 | 5,608 |
| Lao People's Dem. Repub. | 838,800 | 795,654 | 730,583 | 4,436,986 | 1,750 | 1,360 |
| Malaysia | 2,476,673 | 2,329,632 | 2,626,265 | 22,898,437 | 11,415 | 2,237 |
| Marshall Islands | 4,255 | 4,794 | 5,221 | 38,627 | 4,881 | 4,285 |
| Micronesia | 11,570 | 11,557 | 12,461 | 68,872 | 3,608 | 2,678 |
| Mongolia | 337,864 | 278,393 | 219,044 | 2,123,833 | 4,541 | 1,187 |
| Nauruǂ | 888 | 1,001 | 1,090 | 8,062 | 17,196 | 5,608 |
| New Caledoniaǂ | 20,023 | 19,360 | 19,100 | 204,635 | 41,373 | 5,608 |
| Niue*ǂ | 115 | 116 | 120 | 840 | 12,280 | 5,608 |
| Northern Mariana Islands*ǂ | 4,387 | 4,943 | 5,384 | 39,827 | 50,517 | 5,608 |
| Palau* | 1,697 | 1,912 | 2,082 | 15,405 | 11,787 | 6,962 |
| Papua New Guinea | 995,731 | 948,250 | 886,002 | 4,789,338 | 2,090 | 2,207 |
| Philippines | 11,254,961 | 10,651,592 | 10,265,429 | 68,527,413 | 3,681 | 414 |
| Pitcairn Islandsǂ | 5 | 5 | 5 | 34 | 50,517 | 5,608 |
| Samoa | 24,176 | 25,209 | 22,671 | 121,172 | 4,297 | 2,271 |
| Solomon Islands | 81,736 | 78,008 | 70,711 | 353,136 | 1,941 | 1,178 |
| Taiwan | 973,711 | 979,848 | 1,231,288 | 20,196,191 | 50,517 | 7,663 |
| Tokelau*ǂ | 129 | 130 | 134 | 944 | 4,787 | 3,860 |
| Tongaǂ | 12,910 | 13,304 | 12,818 | 67,138 | 4,551 | 5,608 |
| Tuvaluǂ | 953 | 963 | 994 | 6,982 | 6,980 | 5,608 |
| Vanuatu | 34,768 | 33,471 | 28,441 | 167,972 | 3,289 | 3,523 |
| Vietnam | 7,740,845 | 7,221,240 | 6,614,674 | 71,870,842 | 2,005 | 1,340 |
| Wallis and Futuna Islands*ǂ | 1,505 | 1,521 | 1,568 | 11,020 | 4,787 | 3,860 |

* GNI per capita utilized the average GNI from similar countries in the same region (defined by income classification when available or similar economies and industries)

ǂ Minimum wage utilized the average minimum wage from similar countries in the same region (defined by income classification when available or similar economies and industries)
